# *Salmonella* in Coastal Birds in Chile: Detection of a Multidrug-Resistant *S*. Infantis Bearing the *bla*_*CTX-M−65*_ Gene in a pESI-Like Megaplasmid in Humboldt Penguins

**DOI:** 10.1155/2024/1949535

**Published:** 2024-06-10

**Authors:** Clara M. Wiederkehr, Julio Alvarez, Laura Torre-Fuentes, Oscar I. Crespo-Lopez, Paulina Calfucura, Maria Ugarte-Ruiz, Viviana Toledo, Peter W. W. Lurz, Patricio Retamal

**Affiliations:** ^1^ VISAVET Health Surveillance Centre Universidad Complutense de Madrid MadridSpain; ^2^ Departamento de Sanidad Animal Facultad de Veterinaria Universidad Complutense de Madrid MadridSpain; ^3^ Facultad de Ciencias Veterinarias y Pecuarias Universidad de Chile SantiagoChile; ^4^ Servicio Agrícola y Ganadero de Chile Ministerio de Agricultura SantiagoChile; ^5^ Royal (Dick) School of Veterinary Studies and the Roslin Institute University of Edinburgh Edinburgh ScotlandUK

## Abstract

*Salmonella enterica* is one of the most important foodborne pathogens worldwide, and the emergence of multidrug resistance (MDR) clones can aggravate its public health importance. Wildlife species may act as reservoirs of these clones, but their role is not well understood. In this study, faecal samples from shorebirds, with a focus on the endangered Humboldt penguin (*Spheniscus humboldti*), collected from five sites in central Chile with different levels of anthropogenic pressure were analysed to characterize antimicrobial resistant *S. enterica* serovars. Overall, *Salmonella* was isolated from 22 of the 595 samples (3.7%), with positivity ranging between 1.6% and 9.5%, depending on the sampling site. Four of the *Salmonella* isolates were retrieved from Humboldt penguin samples (1.4% positive samples in this species). Serovars Infantis (nine isolates), Typhimurium (six), Goldcoast (four), and Enteritidis, Agona, and Give (one isolate each) were identified. Resistance levels were the highest for sulphamethoxazole (13/21 isolates with a non-wild-type phenotype), ciprofloxacin, tetracycline, and trimethoprim (11/21 each). Whole-genome sequencing performed on eight *S*. Infantis strains revealed that seven carried the plasmid replicon IncFIB (pN55391), indicating the presence of the pESI-like megaplasmid, harbouring resistance determinants to multiple antimicrobial classes as well as heavy metal, biocides, and virulence-related genes. Furthermore, five *S*. Infantis isolates that showed an ESBL phenotype carried the *bla*_*CTX-M−65*_ gene, three of which were detected in Humboldt penguin faeces. The finding of an international emerging *S*. Infantis clone in protected wildlife is of concern to environmental, animal, and public health specialists, supporting initiatives for an active surveillance of resistance and virulence traits in wildlife exposed to anthropogenic areas.

## 1. Introduction

Among the emerging pathogens at the human–animal–environment interface, bacteria with antimicrobial resistance are of particular concern, as they hinder the treatment of human and animal diseases and can facilitate their propagation. This phenomenon is further complicated by the positive selection and horizontal mobility of numerous genes conferring resistant phenotypes between bacterial species, especially in environments under high antimicrobial selective pressure [1]. In addition, this resistance has been identified as a factor favouring cross-species transmission [2]. While this is a natural evolutionary strategy for bacterial survival, the misuse of antibiotics in human and veterinary medicine has increased its frequency. These resistant phenotypes may eventually emerge on a local, regional, or even global scale [3]. In this context, *Salmonella enterica* has been of great concern recently due to the global rise of multi-resistant serovars [4].

*S. enterica* is a gram-negative, multi-host bacterial species of great importance as a foodborne pathogen, producing tens of millions of infections worldwide each year which in certain cases can lead to severe disease [5, 6, 7]. The infection generally occurs by the faecal–oral route, usually through contaminated food or water. However, other routes have been described, such as through vectors (insects, rodents, and birds) or direct contact with or consumption of wild animals [8].

Typically, salmonellosis is self-limiting, and antibiotics are not recommended except in cases of extra-intestinal infections. However, the increasing number of multidrug-resistant (MDR) strains aggravates these cases since they are usually associated with increased morbidity and mortality [9]. As resistance coding genes can be horizontally transmitted between bacterial species [5], the circulation of these resistance determinants threats both animal and public health. The presence of resistant bacteria in wildlife may be a strong indicator of human-derived environmental pollution, demonstrating the complex epidemiology of infection with MDR *Salmonella*.

Among *Salmonella* serovars, *S. enterica* serovar Infantis (*S*. Infantis) is one of the five most frequently isolated serovars in Europe [10] and one of the most frequent in Chile [11, 12]. Currently, it is a serotype of global importance due to the emergence of clones containing megaplasmids known as “plasmid of emerging *S*. Infantis” (pESI) or pESI-like [13], first reported in Israel [14], and soon after in Italy [15], which contain drug resistance genes [16]. Within the emergent pESI-carrying *S*. Infantis clone, strains harbouring the ESBL *bla*_*CTX-M−65*_ gene have been described in several regions of the world, like North America [17, 18], Asia [19, 20], Europe [9], and South America [21, 22, 23], including Chile [24, 25]. This is a troublesome finding since the carriage of this gene, which was first isolated in enterobacteria in 1989 and has since then experienced an exponential increase [17, 18], confers resistance to third generation cephalosporins, which are often used for treatment of invasive *Salmonella* infections [15]. Nowadays, human cases of *Salmonella* Infantis harbouring ESBL genes are mainly associated to consumption of poultry meat, where it has been detected [18, 21, 22, 23], although pESI-like positive *S*. Infantis isolates carrying the *bla*_*CTX-M−65*_ gene have been also found in other animal species including cattle and horses [26].

Even though human infections due to non-typhoidal *Salmonella* are typically linked to livestock/animal products, wildlife can also play a role in the maintenance and spread of MDR *Salmonella* clones, particularly when involving animal species capable of long-distance travels [27]. Furthermore, *Salmonella* can also cause epidemics in wildlife, such as the one described in garden birds in the United Kingdom [28] and Japan [29]. Among waterfowl, ducks and gulls are important reservoir species, as they can maintain the infection in the environment and spread it due to their migratory nature [27, 30]. There are also several reports of *Salmonella* isolation in penguins [31, 32, 33, 34], most of them from Antarctica, suggesting that increased tourism and proximity to human settlements in these areas have an association with *Salmonella* detection [35, 36, 37, 38].

In order to characterize the presence of antimicrobial resistant *Salmonella* strains in coastal wild birds in central Chile, we performed a study involving five sites subjected to different anthropogenic pressure. Environmental faeces were collected from some endangered species, such as the Humboldt penguin (*Spheniscus humboldti*), and other ubiquitous and opportunistic, such as the Dominican gull (*Larus dominicanus*). All bird species sighted during the fieldwork are listed in Table S1.

## 2. Material and Methods

### 2.1. Study Area and Sample Collection

**Figure 1:**
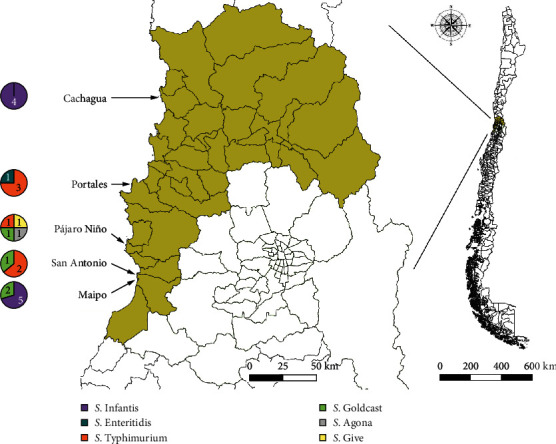
Location of the sampling points in the Valparaíso region (greenish area) and the number of positive samples and serovars identified.

Five sampling points subjected to different anthropogenic influence were selected for this study, all located in the Valparaíso region of Chile (Figure 1). The exact location and description of these points is explained in Table S2.

The fieldwork for sampling was conducted between October and December 2022, at the end of the breeding season for Humboldt penguins, to not interfere with their reproduction. In addition, this is the time when migratory birds, intercontinental, such as the Ruddy turnstone (*Arenaria interpres*), the Franklin's gull (*Leucophaeus pipixcan*), the sanderling (*Calidris alba*), the black skimmer (*Rynchops niger*) or the whimbrel (*Numenius phaeopus*), and latitudinal such as the Peruvian pelican (*Pelecanus thagus*), the black vulture (*Coragyps atratus*), or the turkey vulture (*Cathartes aura jota*), arrive [39, 40, 41]. Therefore, this season was selected to maximize the number of individuals/species that could be present in the area. The number of penguin nests sampled on Cachagua Island were 243, a 44.4% of the total number of nests counted on the island. Likewise, on Pájaro Niños Islet, the sampled nests were 41, an 89.13% of the nest counted.

**Figure 2:**
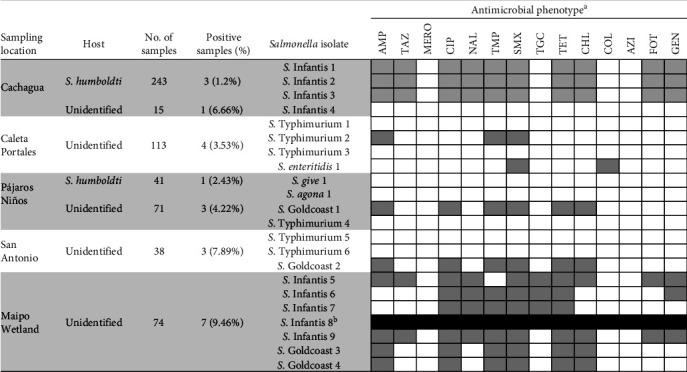
Number of samples collected and *Salmonella*-positive samples per location, serotypes, and resistance profile. ^a^AMP, ampicillin; AZI, azitromicine; CHL, chloramphenicol; CIP, ciprofloxacin; COL, colistin; FOT, cefotaxime; GEN, gentamicin; MERO, meropenem; NAL, nalidixic acid; SMX, sulfametoxazol; TAZ, ceftazidime; TET, tetracycline; TGC, tigecycline; TMP, trimethoprim; results for azythromycin and meropenem (wild-type phenotype in all isolates) not shown. ^b^Antimicrobial resistance data not available. White and grey cells indicate a wild-type and non-wild-type phenotype, respectively.

Environmental fresh faeces (*n* = 595) were collected using sterile cotton swabs (one swab per sample) from the ground or from penguin nests (Figure 2).

### 2.2. Isolation and Identification of *Salmonella*

Samples were immediately immersed in a sterile glass tube containing 5 mL of buffered peptone water (Difco® APT broth) and transported at environmental temperature on the same day to the laboratory. Once there, tubes were incubated at 37°C for 24 hr. Then, 100 *µ*L of this broth was inoculated into Modified Semi-Solid Rappaport-Vassiliadis (MSRV) medium supplemented with 20 *µ*g/mL novobiocin. After an incubation for 24–48 hr at 41.5°C, bacteria were seeded into xylose lysine deoxycholate (XLD) agar plates and incubated for 24 hr at 37°C.

Suspected *Salmonella* colonies were subjected to a PCR for detection of the *invA* gene as previously described [42], and *S*. *enterica* isolates were serotyped according to the Kauffman–White scheme [43] at the national reference laboratory of the Livestock and Agriculture Service.

### 2.3. Antimicrobial Susceptibility Typing

Isolates were submitted to the VISAVET Health Surveillance Centre for antimicrobial susceptibility typing (AST) using the microdilution method. Briefly, minimal inhibitory concentrations (MIC) were determined using the twofold broth microdilution reference method, according to ISO 20776-1 : 2021 using EUVSEC3 and EUVSEC2 Sensititre plates (Trek Diagnostic Systems; Thermo Scientific, Waltham, MA, USA). Panel EUVSEC2 was only used when resistance to cefotaxime, ceftazidime, and/or meropenem was observed, following Commission Implementing Decision 2020/1729/EU. The panel EUVSEC3 included amikacin, ampicillin, azithromycin, cefotaxime, ceftazidime, chloramphenicol, ciprofloxacin, colistin, gentamicin, meropenem, nalidixic acid, sulfamethoxazole, tetracycline, tigecycline, and trimethoprim. Besides, panel EUVSEC2 included cefepime, cefotaxime, cefotaxime/clavulanic acid, cefoxitin, ceftazidime, ceftazidime/clavulanic acid, ertapenem, imipenem, meropenem, and temocillin (Table S3). Interpretation of quantitative data was performed as described by Commission Implementing Decision 2020/1729/EU, as well as the European Committee on Antimicrobial Susceptibility Testing EUCAST using epidemiological cutoff values (ECOFF) to classify isolates as wild-type or non-wild-type as previously recommended [44].

### 2.4. Whole-Genome Sequencing and Bioinformatic Analysis of *S*. infantis Isolates

All isolates belonging to the Infantis serovar were subjected to whole-genome sequencing (WGS). Bacterial genomic DNA was purified from axenic cultures using Qiagen DNA Blood and Tissue Kit, following the manufacturer's instructions, and quantification of the DNA concentration was done using a Qubit® fluorometer (Invitrogen). WGS libraries were prepared from 1 ng of bacterial DNA by using the Nextera XT DNA Library Preparation Kit (Illumina) following manufacturer instructions. The concentrations of each library were adjusted to 4 nM to obtain equimolar DNA concentrations in a single pool of libraries and sequenced in a MiSeq platform using the 2 × 300 cycle V3 Kit (Illumina).

Illumina raw reads were filtered and trimmed using Trimmomatic [45]. Once the reads passed the quality control with FastQC, genomes were assembled by SPAdes [46], and QUAST [47] was used to evaluate the quality of the assemblies. Serotyping analysis was performed using SISTR [48] for *Salmonella*. Multilocus sequence typing (MLST) was performed to assign MLST profiles to assemblies by mlst software (Seemann, https://github.com/tseemann/mlst). Assemblies were screened by Resfinder [49] using an identity and coverage threshold of >80% for the presence of antimicrobial resistance genes. Plasmid replicons were identified using PlasmidFinder [50] and MOB-suite [51] with an identity and coverage threshold of 80%. PointFinder [52] was used for the identification of the point mutations. Virulence genes were identified by Abricate (Seemann, https://github.com/tseemann/abricate; [53]) and AMRFinder [54] using an identity and coverage threshold of >90% and >80%, respectively. IntegronFinder [55] was used for the detection of integrons. The presence of integrases was confirmed by BLAST using *intl1* (ANE23618.1) and *intl2* (MK994977.1) as the reference sequences.

The raw reads generated in this study were deposited in the European Nucleotide Archive (PRJEB70652).

### 2.5. Phylogenetic Analysis of *S*. Infantis Isolates

**Figure 3:**
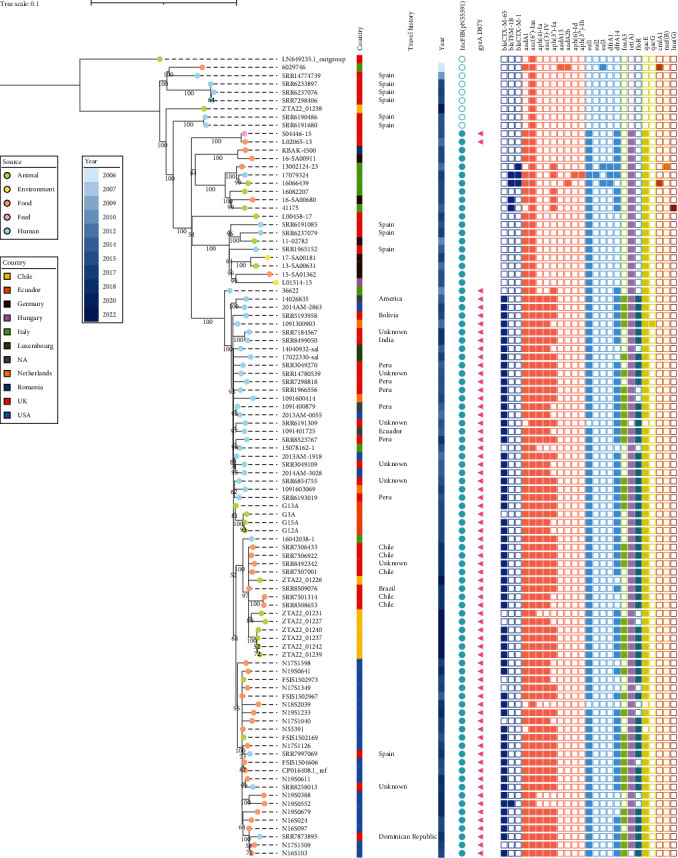
Maximum likelihood phylogenetic tree of 95 *S*. Infantis isolates. The major external nodes are labelled with circles (green, yellow, orange, pink, and blue) indicating the source of each strain. The following information is presented to the right of the isolate IDs: country of origin, information of travel history (if available), presence of the pESI-like plasmid, presence of *gyrA* D87Y point mutation, and presence/absence of ARGs. Bootstrap values (>50 support) are shown in the tree.

The eight *S*. Infantis isolates identified here (see below) and 87 external isolates described in previous studies retrieved from animals, environment, food, feed, and humans (Figure 3) and selected based on their genetic heterogeneity (e.g., located in different regions of previously built phylogenetic trees) [13, 18, 56, 57, 58] were included in the phylogenetic analysis (Table S4). All reads were mapped against the genome of *S*. Infantis (Genbank Accession Number CP016408.1) [58], used as a reference, by BWA [59] with default parameters. SAMtools [60] was used to sort and compressed the resulting SAM files into BAM files. Variant calling was then performed by BCFtools [61] applying “mpileup” and “call” options and excluding SNPs with a base quality <30 and a mapping quality <30. Prophage regions for the reference genome were identified by PHASTER (PHAge Search Tool) [62, 63]. Consensus sequences were then generated by BCFtools masking the identified prophage regions.

Concatenated consensus sequences were used to build a maximum likelihood phylogenetic tree in RAxML [64] using the general time-reversible substitution evolutionary model with gamma correction and 1,000 bootstrap replicates. The tree was rooted using a *S*. Infantis sequence from a strain isolated in 1973 in the United Kingdom (Genbank Accession Number LN649235.1) [65] and visualized using iTOL editor [66].

## 3. Results

### 3.1. *Salmonella* Detection

A total of 595 samples were collected from shorebirds, 284 of which belonged to faeces collected in Humboldt penguin nests, and the rest from unidentified birds, most of which would correspond to pelicans and gulls (Figure 2). *Salmonella* was detected in 22 samples (3.7%), of which four were from penguins (percentage of positives of 1.4% in this species) and the rest from other birds (18/311, 5.8%) (Figure 2).

The highest proportion of *Salmonella*-positive samples was obtained in the Maipo River Wetland (9.5%). In the rest of the areas, *Salmonella* detection ranged from 1.6% to 7.9% of the samples.

Overall, six serovars were identified, with Infantis being the most common (9/22, detected in two of the five locations), followed by Typhimurium (6/22, three locations) and Goldcoast (4/22, three locations). All three *Salmonella* isolates from penguin nests in Cachagua Island were *S*. Infantis, while the remaining isolate retrieved from pinguin faeces originated from Pájaros Niños Islet and was *S*. Give (Figure 1).

### 3.2. Antimicrobial Resistance Phenotypes

The resistance profile was determined in 21/22 *Salmonella* (all except one *S*. Infantis from Maipo that could not be retrieved after its shipment to the VISAVET centre) (Figure 2). Non-wild-type phenotypes to the antimicrobials included in panel 1 (EUVSEC3) involved most commonly sulfamethoxazole (13/21 isolates) followed by ciprofloxacin, tetracycline and trimethoprim (11/21), ampicillin (10/21), and chloramphenicol (9/21). Proportion of isolates with non-wild-type phenotypes to other antimicrobials ranged from 5% to 33% except for azithromycin and meropenem (wild-type phenotype in all isolates) (Figure 2). When considering the phenotypes depending on the serovar, *S*. Infantis isolates were the most resistant, with non-wild-type phenotypes to between 6 and 10 antimicrobials, except for one pansusceptible isolate retrieved from Isla Cachagua (Figure 2). Five of the eight *S*. Infantis isolates with AST data yielded a non-wild-type phenotype for ceftazidime and cefotaxime and were therefore subjected to AST using panel EUVSEC2; of these, all five showed an ESBL phenotype, one of which also had an AmpC phenotype (Tables 1 and S3).

Regarding the remaining serovars, all four *S*. Goldcoast isolates had the same profile of resistance to six antibiotics (AMP, CHL, CIP, TET, TMP, and SMX), while most isolates belonging to serovars Typhimurium, Give, Agona, and Enteritidis were pansusceptible (Figure 2). Altogether, isolates belonging to serovars for which more than one isolate was analysed (Infantis, Typhimurium, and Goldcoast) had very similar resistance phenotypes (Figure 2),

### 3.3. *S*. Infantis Genomic Analyses

Different genes conferring resistance to aminoglycosides, beta-lactams, phenicol, sulphonamide, tetracycline, disinfectant, fosfomycin, and trimethoprim were found in seven of the eight *S*. Infantis isolates analysed. The pansusceptible strain from Isla Cachagua harboured the cryptic *aac* (*6′*)-*Iaa* gene only. The extended-spectrum beta-lactamase-encoding gene *bla*_*CTX-M−65*_ was present in five strains, four of which carried also a set of 10 additional resistance genes. The five *bla*_*CTX-M−65*_-positive isolates with an ESBL phenotype were retrieved from three Humboldt penguins and two unidentified coastal birds. In addition, *parC* T57S and *gyrA* D87Y mutations in the quinolone resistance determinant regions (QRDR) were found in all isolates except the pansusceptible strain, which only presented the mutation in *parC* (Table 1).

Seven of the eight isolates carried the plasmid replicon IncFIB (pN55391), indicative of the presence of the pESI-like plasmid, with the pansusceptible strain being again the outlier. All eight isolates carried the stress response genes *golT*, *golS*, and *arsR* as well as the virulence genes *iroB*, *iroC*, and *sinH*. However, strains carrying the pESI-like plasmid also harboured other virulence (*ybtP*, *ybtQ*, *ybtE*, *ybtT*, *ybtU*, *ybtA*, *ybtX*, *ybtS*, *irp1*, *irp2*, and *fyuA/psn*), heavy metals (*merR*, *merT*, *merP*, and *mer*C), and biocide (*qacEdelta1*) resistance genes (Table 1).

In addition, class 1 integrons were identified in the pESI-positive isolates, while class 2 integrons were found in four of the five strains harbouring *bla*_*CTX-M−65*_ and one pESI-positive strain lacking the ESBL gene (Table 1).

All antimicrobial resistance and virulence genes and mobile genetic elements are shown in the supplementary material (Table S4).

### 3.4. Phylogenetic Analysis

*In silico* serotyping and MLST identified all *S*. Infantis retrieved in this study as ST32. The phylogenetic analysis including 87 *S*. Infantis isolates from other studies separated those with and without the pESI-like plasmid in two different clades as expected. Strains carrying pESI typically carried a higher number of ARGs, with a subclade containing only isolates with the *gyrA* D87Y mutation, most of which (59/70, 84.3%) also carried the *bla*_*CTX-M−65*_, in which all the pESI-like positive isolates retrieved in this study were included. The pESI-positive isolates from this study were distributed in three different parts of this subclade. Four of the five isolates with *bla*_*CTX-M−65*_, separated by 3–6 SNPs, clustered together and were separated from the two strains lacking *bla*_*CTX-M−65*_ by 29–37 SNPs. These two isolates lacking the ESBL gene differed by 31 SNPs. Lastly, the last *bla*_*CTX-M−65*_-positive strain lacking the class 2 integron was 27 SNPs away from the closest Chilean isolate, one of the four *bla*_*CTX-M−65*_-positive strains clustered together. Regarding proximity to external strains, the closest neighbours included food isolates from Chile, Brazil, and of unknown origin, at a minimum distance of 13 SNPs (Figure 3).

## 4. Discussion

*Salmonella* is a widely distributed and multi-host food pathogen, leading to millions of infections per year. This makes salmonellosis one of the most important foodborne diseases worldwide and one of the leading causes of foodborne outbreak-associated deaths [5, 11].

Even though animal-derived foods are considered the traditional reservoir of *Salmonella*, there are reports of its direct transmission between humans and/or domestic animals and wildlife [28, 29, 67, 68, 69]. A previous study in Chile already demonstrated the circulation of *S*. Enteritidis strains collected from gulls located in the same area studied here which were closely related to isolates from poultry and humans, providing evidence of the potential role of coastal birds in the epidemiology of this serovar in the human–animal interface [70, 71]. In addition, many studies have reported a rise in the risk of *Salmonella* infection in wildlife related to an increased contact with humans, suggesting that the presence of this bacterium can serve as a marker of anthropogenic influence [35, 36, 37, 38, 72, 73].

The prevalence of *Salmonella* in animal populations is often quite variable. In seabirds, especially in gulls of various species, *Salmonella* detection ranges from 9% in California [74] to 9.2%–17% in western Europe, [75, 76, 77] and 30% in Australia [78]. Previous studies in Chile have reported positive detection ranging from 2.3% to 25% [79, 80, 81]. In the specific case of penguins, *Salmonella* detection is even less common worldwide, with most of the studies reporting absence of infection [12, 32, 35, 82, 83, 84, 85, 86] or infection levels below 4% [31, 33, 38, 83, 86]. Higher values have been reported from Bird Island, South Georgia (6.67%) [84], and Ross Island, Antarctica (13.5%) [32]. Our results are consistent with the large variability of *Salmonella* detection.

The most commonly reported serovars in animal-derived foods and human cases are *S*. Enteritidis, *S*. Typhimurium, and the emergent MDR *S*. Infantis [11, 87, 88]. While there is limited information available on penguins, various serotypes have been documented in Chile [31] and the adjacent areas of Argentina [89] and Antarctica [33, 36, 38, 84, 86], suggesting that Enteritidis and Typhimurium are commonly occurring serotypes in these bird species. In contrast, the most frequent serovar found in this study was *S*. Infantis (9/22), which is a remarkable finding, since it is a highly prevalent serovar in broiler production worldwide [90]. The recent emergent MDR clone carrying multiple resistance and virulence genes in a plasmid (pESI-like) has been a cause of concern due to its widespread distribution in poultry populations in several countries [87]. Furthermore, in wildlife, this MDR *S*. Infantis clone had only been described once in Chile in 2018, in an Magellanic horned owl (*Bubo magellanicus*). The animal, which was admitted to a recovery centre in southern Chile, carried an isolate harbouring the *bla*_*CTX-M−65*_ gene in a IncFIB plasmid [24]. In this study, five *S*. Infantis isolates were retrieved from two locations separated by 115 km (Maipo Wetland and Cachagua Island) and carried the IncFIB pESI-like plasmid and the *bla*_*CTX-M−65*_ ESBL gene. Moreover, isolates were phylogenetically related (<43 SNPs) to other strains retrieved in England and Wales from food imported from Chile and Brazil [13]. This could indicate that *bla*_*CTX-M−65*_-positive *S*. Infantis strains, similar to those described in other countries in America [18, 26, 58, 91] and Europe [13, 15, 56, 92], are also circulating in Chile. This is also supported by the description of phenotypically MDR *S*. *infanti*s strains carrying the *bla*_*CTX-M−65*_ gene isolated from chicken meat samples of retail [25], through the route of exposure of the coastal bird populations here is unclear. In this regard, the infection could have occurred through contaminated water, since several studies have shown the risk of water pollution with *Salmonella* associated to human outbreaks [93, 94, 95], a phenomenon that could lead to a higher prevalence of antibiotic resistance in birds [70, 96]. Given that the areas of this study are in the vicinity of outfalls that discharge wastewater into the sea, there is a risk of *Salmonella* dispersion from human sources to wildlife populations. This could explain the similarity between the *bla*_*CTX-M−65*_-carrying isolates retrieved from Cachagua Island (<6 SNPs) (Figure 3), which has a submarine outfall in Quintero, 40 km away from the sampling site [97]. Another possibility is the bacterial spreading through infected shorebirds, especially by omnivorous and opportunistic species, such as gulls, as previously described [98, 99]. This in turn could explain the great similarity (<4 SNPs) of *bla*_*CTX-M−65*_-positive isolates from points as far apart as Cachagua and Maipo Wetland.

The persistence of the MDR *S*. Infantis strains in the sampled areas could be then favoured by the presence of copper refineries and oil and gas platforms in the surrounding bays, such as Quintero Bay, which lead to high levels of air and sea pollution, given the well-established association between genes conferring resistance to heavy metals and antimicrobials, which would allow bacteria to thrive in this environment [100, 101, 102]. This hypothesis would be supported by the detection of the *merR*, *merT*, *merP*, and *merC* genes, involved in resistance to mercury, in isolates carrying the pESI-like plasmid (Table S4).

As for the other serovars, Typhimurium was the second most common serovar, in agreement with the literature cited above, but in contrast, Enteritidis was rare, and Goldcoast was more abundant (Figures 1 and 2). Furthermore, the Typhimurium isolates retrieved in this study were mostly pansusceptible, with only one isolate showing a non-wild-type phenotype to ampicillin, sulfamethoxazole, and trimethoprim. This is in contrast with the typical resistance patterns in isolates retrieved from animals and humans described in other regions, including Europe [103] and the USA, which usually involve ampicillin, chloramphenicol, streptomycin, sulphonamides, and tetracycline [104], or Chile, involving ciprofloxacin and nalidixic acid [105]. The resistance pattern found in the only *S*. Enteritidis isolated recovered (non-wild-type phenotype for colistin and sulfamethoxazole) was partially similar to the ones previously described for isolates belonging to this serovar retrieved from gulls from the same area (which were resistant to sulfamethoxazole but also to streptomycin, tetracycline, gentamicin, and ampicillin) [70, 71]. However, further studies would be needed in order to assess their genetic relatedness.

Regarding *S*. Goldcoast, this serovar has been described mainly in outbreaks in Europe [106, 107] and Asia, with the emergence of MDR clones [108, 109]. This is consistent with the resistance phenotype observed here, since all isolates had a non-wild-type phenotype for more than six antimicrobial classes. Interestingly, *goldcoast* is a rare serovar in the country, and had not been previously described in studies in wildlife in Chile [79], so further studies are needed to identify the likely origin of these isolates, found in three of the five locations sampled.

Also noteworthy is the presence of *S*. Give in the penguin isolate from Pájaro Niños islet. *S*. Give is an uncommon serovar, which has been mostly isolated from pigs and cattle in some outbreaks in Europe and North America. Although rare in humans, cases of *S*. Give are associated with increased virulence and severity of diseases, which translates into higher rates of hospitalization [110, 111, 112, 113, 114]. There is one report of isolation of this serovar in South America, in paediatric patients in Venezuela [115] and reports in wild birds are occasional, with a description in crows in the USA [116], and an ostrich in Canada [117].

Finally, one isolate belonging to the Agona serovar was found (Figure 1), a serovar previously retrieved in Chile from different sources, including poultry and pigs from backyard production systems [118], wild birds such as penguins and seagulls [31, 79], and humans [119], although most of these isolates were resistant to antimicrobials unlike the isolate found here.

Regarding the limitations of this study, samples were collected only once, and thus, our detection rates estimates would not be representative of the year [116, 120]. The approach chosen here to minimize disturbance of animals was based on collecting faeces from the environment (nests or soil). Subsequently, samples were not assigned to particular individuals, and variables such as age or sex, which are thought to impact the risk of *Salmonella* infection in birds, were not taken into account [28, 121]. Nevertheless, and despite the number of samples collected was limited, our results provide evidence of the presence of multiple *S. enterica* serovars, including MDR isolates belonging to an international emerging *S*. Infantis clone in the sampling sites included here irrespective of their different characteristics. Additional studies including isolates from potential sources (livestock, food, and human cases along with the environment) would be needed to identify the most likely source of infection for coastal birds in the sampling areas and design possible preventive and mitigation measures aiming at decreasing the exposure of wildlife to MDR *Salmonella* in the region and the potential risks this may cause for humans in the future. Further analysis using high-resolution characterization techniques on the *Salmonella* isolates belonging to other serovars found here would also help to assess whether the same strains are shared between sampling locations as suggested by the similar resistance phenotypes found not only for Infantis but also for Typhimurium and Goldcoast.

## Figures and Tables

**Table 1:** Presence of plasmid replicons, antimicrobial resistance determinants (ARGs), integrases, and *gyrA* point mutations detected in *S*. Infantis isolates from Chile^a^.

Isolate	Location	Hosts	Phenotype	IncFIB	*bla_CTX-M-65_*	Other ARGs	*intI1*	*intI2*	*gyrA*
ZTA22/01226	Maipo Wetland	Unidentified	ESBL + AmpC	X	X	*aadA1*, *aph(4)-Ia*, *aac(3)-IV*, *aph(3′)-Ia*, *floR*, *sul1*, *tet(A)*, and *qacE*	X	—	X
ZTA22/01242			ESBL	X	X	*aac(6′)-Iaa, aadA1, aph(4)-Ia, aac(3)-IV, aph(3′)-Ia, floR, sul1, tet(A), qacE, fosA3, and dfrA14*	X	X	X
ZTA22/01227			—	X	—	*aac(6′)-Iaa*, *aadA1*, *aph(4)-Ia*, *aac(3)-IV*, *sul1*, *tet(A)*, *qacE*, *fosA3*, and *dfrA14*	X	X	X
ZTA22/01231			—	X	—	*aac(6′)-Iaa*, *aadA1*, *sul1*, *tet(A)*, *qacE*, and *dfrA14*	X	—	X
ZTA22/01237	Cachagua	*Spheniscus humboldti*	ESBL	X	X	*aac(6′)-Iaa*, *aadA1*, *aph(4)-Ia*, *aac(3)-IV*, *aph(3′)-Ia*, *floR*, *sul1*, *tet(A)*, *qacE*, *fosA3*, and *dfrA14*	X	X	X
ZTA22/01239			ESBL	X	X	*aac(6′)-Iaa*, *aadA1*, *aph(4)-Ia*, *aac(3)-IV*, *aph(3′)-Ia*, *floR*, *sul1*, *tet(A)*, *qacE*, *fosA3*, and *dfrA14*	X	X	X
ZTA22/01240			ESBL	X	X	*aac(6′)-Iaa*, *aadA1*, *aph(4)-Ia*, *aac(3)-IV*, *aph(3′)-Ia*, *floR*, *sul1*, *tet(A)*, *qacE*, *fosA3*, and *dfrA14*	X	X	X
ZTA22/01238		*Unidentified*	—	—	—	*aac(6′)-Iaa*	—	—	—

^a^The *parC* T57S mutation was found in all isolates and therefore was excluded from the table.

## Data Availability

The raw reads generated in this study were deposited in the European Nucleotide Archive (PRJEB70652).

## Supplementary Materials

Supplementary Materials Table S1: birds sighted on sampling days at each point. Table S2: location of sampling points and brief description. Table S3: minimum inhibitory concentration and ENA references for the eight Salmonella Infantis sequenced in this study. Table S4: biological and genotypic information of the S. Infantis strains used in the phylogenetic study.
