# Supplementary material for: Salmonella in Coastal Birds in Chile: Detection of a Multidrug-Resistant S. Infantis Bearing the blaCTX-M−65 Gene in a pESI-Like Megaplasmid in Humboldt Penguins
Source: Transbound Emerg Dis. 2024 Jun 10;2024:1949535. doi: 10.1155/2024/1949535 (PMC12020390; doi:10.1155/2024/1949535)
Supplement: Supplementary Materials — Table S1: birds sighted on sampling days at each point. Table S2: location of sampling points and brief description. Table S3: minimum inhibitory concentration and ENA references for the eight Salmonella Infantis sequenced in this study. Table S4: biological and genotypic information of the S. Infantis strains used in the phylogenetic study. [file 1949535.f1.pdf]

Supplementary Material

Table S1. Birds sighted on sampling days at each point:

**Sampling location**	**Possible hosts (birds sighted)**
Cachagua	*Sphenscus humboldti*<br>*Sula variegata*<br>*Pelecanus thagus*<br>*Coragyps atratus*<br>*Cathartes aura*<br>*Phalacrocprax brasilianus brasilianus*<br>*Haematopus ater*<br>*Haematopus paliatus*
Caleta Portales	*Larosterna inca*<br>*Pelecanus thagus´*<br>*Phalacrocprax brasilianus brasilianus*<br>*Larus dominicanus*<br>*Pelecanus thagus*
Pájaro Niños	*Sphenscus humboldti*<br>*Haematopus ater*<br>*Arenaria interpres*<br>*Larus dominicanus*<br>*Pelecanus thagus*<br>*Calidris bairdis*<br>*Calidris alba*
San Antonio	*Larus dominicanus*<br>*Larosterna inca*<br>*Pelecanus thagus*<br>*Columba livia*
Maipo Wetland	*Phalacrocprax brasilianus brasilianus*<br>*Larus dominicanus*<br>*Haematopus palliates*<br>*Numenius phaeopus*<br>*Larosterna inca*<br>*Vanellus chilensis chilensis*<br>*Leucophaeus pipixcan*<br>*Cathartes aura*<br>*Haematopus paliatus*<br>*Numenius phaeopus*<br>*Anas georgica spinicauda*<br>*Rynchops niger*<br>*Larosterna inca*<br>*Himanyopus mexicanus melanurus*

Table S2. Location of sampling points and brief description.

**Sampling point**	**Coordinates**	**Brief description**
Isla Cachagua	32º 35’ S, 71º 27’ W	An island of 4.5 hectares about 100 metres from the continental coast that can only be accessed for research purposes with the authorisation of the National Forestry Corporation (CONAF). It is one of the most important nesting sites for Humboldt penguins (*Spheniscus humboldti*) in central Chile, with a population that usually ranges between 400 and 750 breeding pairs. The number of active nests at the last count, carried out on the same day as the samples were taken, was 547.
Caleta Portales	33º 1' S, 71º 35' W	Located in the north of the Valparaíso region, this is a tourist spot famous for local fishing where several bird species coexist with a large population of sea lions (*Otaria flavescens*) and abandoned dogs, where food markets in poor hygienic conditions are located.
Pájaro Niños Islet	33º 21’ S, 71º 41’ W	An island of about 3.4 hectares, separated 150 metres from the continental coast. There are currently 46 active nests of Humboldt penguin. Also protected, but the population has been drastically reduced in last years, as the island was joined to the mainland by an artificial arm of land, increasing anthropogenic activities and the entry of invasive species such as rats and dogs.
San Antonio	33º 34' S, 71º 36' W	Located 3 km north of the mouth of the Maipo River, it is the port with the largest foreign trade cargo in the country, and the ninth largest in Latin America. As in Caleta Portales, shorebirds and sea lions predominate, living together in overcrowded and in poor hygienic conditions.
Maipo River Wetland	33º 37' S, 71º 37' W	A total of 132 bird species have been recorded in this ecosystem, which has been designated as a "site of regional importance". The wetland is located at the mouth of the Maipo river, the basin of which drains the Metropolitan Region, including the capital city of Santiago.

Table S3

Strain	Biosample	un_accession_numb	Host_species	Sampling_location	TMPCMI	SMXCMI	CIPCMI	TETCMI	MEROCMI	AZICMI	NALCMI	CHLCMI	FOTCMI
ZTA22/01226	SAMEA114776265	ERR12341384	Coastal birds	Maipo River Wetland	≤0,25	>512	0,5	>32	≤0,03	8	>64	>64	>4
ZTA22/01227	SAMEA114776266	ERR12341385	Coastal birds	Maipo River Wetland	>16	>512	0,5	>32	≤0,03	16	>64	≤8	0,5
ZTA22/01231	SAMEA114776267	ERR12341386	Coastal birds	Maipo River Wetland	>16	>512	0,5	>32	≤0,03	16	>64	16	0,5
ZTA22/01237	SAMEA114776268	ERR12341387	Humboldt penguin	Isla Cachagua	>16	>512	0,25	>32	≤0,03	8	>64	>64	>4
ZTA22/01238	SAMEA114776269	ERR12341388	Coastal birds	Isla Cachagua	≤0,25	32	0,03	≤2	≤0,03	8	≤4	≤8	≤0,25
ZTA22/01239	SAMEA114776270	ERR12341389	Humboldt penguin	Isla Cachagua	>16	>512	0,25	>32	≤0,03	4	>64	>64	>4
ZTA22/01240	SAMEA114776271	ERR12341390	Humboldt penguin	Isla Cachagua	>16	>512	0,25	>32	≤0,03	4	>64	>64	>4
ZTA22/01242	SAMEA114776272	ERR12341391	Coastal birds	Maipo River Wetland	>16	>512	0,25	>32	0,06	4	>64	>64	>4

antibiotics panel 1: AMP, ampicillin;
AZI, azitromicine;
CHL, chloramphenicol;
CIP, ciprofloxacin;
COL, colistin;
FOT, cefotaxime;
GEN, gentamicin;
MERO, meropenem;
NAL, nalidixic acid;
SMX, sulfametoxazol;
TAZ, ceftazidime;
TET, tetracycline;
TGC, tigecycline;
TMP, trimethoprim;

antibiotics panel 2: FOX, cefoxitin;
ETP, ertapenem;
IMI, imipenem;
MERO2, meropenem;
TAZ2, ceftazidime;
FEP, cefepime;
F/C, cefotaxime + clavulanic acid;
T/C, ceftazidime + clavulanic acid;
FOT, cefotaxime,
TRM, temocillin

Table S3

Strain	TGCCMI	TAZCMI	COLCMI	AMPCMI	GENCMI	FOXCMI	ETPCMI	IMICMI	MERO2CMI	TAZ2CMI	FEPCMI	F/CCMI	T/CCMI	FOT2CMI	TRMCMI
ZTA22/01226	1	4	≤1	>32	16	16	0,03	≤0,12	≤0,03	4	8	0,25/4	1/4	>64	16
ZTA22/01227	1	1	≤1	4	4										
ZTA22/01231	1	1	≤1	4	≤0,5	32	≤0,015	0,25	≤0,03	1	0,5	0,5/4	1/4	0,5	16
ZTA22/01237	0,5	8	≤1	>32	16	4	≤0,015	0,25	≤0,03	4	8	0,12/4	0,5/4	>64	8
ZTA22/01238	0,5	0,5	≤1	2	≤0,5										
ZTA22/01239	≤0,25	4	≤1	>32	8	8	≤0,015	0,25	≤0,03	4	16	0,12/4	0,25/4	>64	8
ZTA22/01240	≤0,25	4	≤1	>32	8	8	≤0,015	0,25	0,06	4	8	0,12/4	0,5/4	>64	8
ZTA22/01242	≤0,25	4	≤1	>32	8	8	≤0,015	0,25	0,06	8	8	0,12/4	0,25/4	>64	8

antibiotics panel 1: A

Table S4

Strain	Accession_number	Source	Sample_type	Host_species	Sampling_location	Country	Year	Foreign_travel	Source_article	IncFIB(pN55391)	IncI1-I(Alpha)	gyrA_D87Y	parC_T57S
ZTA22/01238	ERR12341388	Animal	Faeces	Coastal birds	Isla Cachagua	Chile	2022	NA	This study	0	0	0	1
06029746§	ERR1014111	Food	Food	Meat from broilers	NA	Italy	2006	NA	Alba et al., 2020	0	1	0	1
SRR6190486	SRR6190486	Human	Faeces	Human	NA	UK	2015	Spain	Lee et al., 2021	0	1	0	1
SRR6191680	SRR6191680	Human	Faeces	Human	NA	UK	2015	Spain	Lee et al., 2021	0	0	0	1
SRR7298406	SRR7298406	Human	Faeces	Human	NA	UK	2016	Spain	Lee et al., 2021	0	0	0	1
SRR6233897	SRR6233897	Human	Faeces	Human	NA	UK	2016	Spain	Lee et al., 2021	0	0	0	1
SRR6237076	SRR6237076	Human	Faeces	Human	NA	UK	2017	Spain	Lee et al., 2021	0	0	0	1
SRR14774739	SRR14774739	Human	Faeces	Human	NA	UK	2010	Spain	Lee et al., 2021	0	0	0	1
ZTA22/01226	ERR12341384	Animal	Faeces	Coastal birds	Maipo River Wetlan	Chile	2022	NA	This study	1	0	1	1
ZTA22/01227	ERR12341385	Animal	Faeces	Coastal birds	Maipo River Wetlan	Chile	2022	NA	This study	1	0	1	1
ZTA22/01231	ERR12341386	Animal	Faeces	Coastal birds	Maipo River Wetlan	Chile	2022	NA	This study	1	0	1	1
ZTA22/01237	ERR12341387	Animal	Faeces	Humboldt penguin	Isla Cachagua	Chile	2022	NA	This study	1	0	1	1
ZTA22/01239	ERR12341389	Animal	Faeces	Humboldt penguin	Isla Cachagua	Chile	2022	NA	This study	1	0	1	1
ZTA22/01240	ERR12341390	Animal	Faeces	Humboldt penguin	Isla Cachagua	Chile	2022	NA	This study	1	0	1	1
ZTA22/01242	ERR12341391	Animal	Faeces	Coastal birds	Maipo River Wetlan	Chile	2022	NA	This study	1	0	1	1
1091603069	ERR2209370	Human	Human	Human	NA	Netherlands	2017	NA	Alba et al., 2020	1	0	1	1
1091600414	ERR2209376	Human	Human	Human	NA	Netherlands	2016	NA	Alba et al., 2020	1	0	1	1
1091300903	ERR2209379	Human	Human	Human	NA	Netherlands	2013	NA	Alba et al., 2020	1	0	1	1
1091400879	ERR2209381	Human	Human	Human	NA	NA	2014	Peru	Alba et al., 2020	1	0	1	1
1091401725	ERR2209388	Human	Human	Human	NA	NA	2014	Ecuador	Alba et al., 2020	1	0	1	1
17079324	ERR2209402	Human	Human	Human	NA	Italy	2017	NA	Alba et al., 2020	1	1	0	1
16066439	ERR2209412	Animal	Animal	Broiler chicken	NA	Italy	2016	NA	Alba et al., 2020	1	0	0	1
16042038-1	ERR2209469	Human	Human	Human	NA	Italy	2016	NA	Alba et al., 2020	1	0	1	1
17022330-sal	ERR2209489	Human	Human	Human	NA	Luxembourg	2017	NA	Alba et al., 2020	1	0	1	1
14040932-sal	ERR2209492	Human	Human	Human	NA	Luxembourg	2014	NA	Alba et al., 2020	1	0	1	1
16082207	ERR2209609	Animal	Animal	Broiler chicken	NA	Italy	2016	NA	Alba et al., 2020	1	0	0	1
13002124-23§	ERR2209663	Food	Food	Meat from pigs	NA	Italy	2013	NA	Alba et al., 2020	1	0	0	1
36622§	ERR2209682	Human	Human	Human	NA	Italy	2011	NA	Alba et al., 2020	1	0	1	1
41175	ERR2209704	Animal	Animal	Broiler chicken	NA	Italy	2014	NA	Alba et al., 2020	1	0	0	1
15078162-1	ERR3562263	Human	Human	Human	NA	Italy	2015	NA	Alba et al., 2020	1	0	1	1
L00458-17	ERR2231067	Animal	Animal	Horse	NA	United Kingdom	2017	NA	Alba et al., 2020	1	0	0	1
L01514-15	ERR2231037	Environment	Environment	Chicken-unspecified	NA	Hungary	2015	NA	Alba et al., 2020	1	0	0	1
S04446-15	ERR2231049	Feed	Feed	Other_Veg_Mineral	NA	United Kingdom	2015	NA	Alba et al., 2020	1	0	1	1
L02065-13	ERR2231046	Food	Food	Meat products from broiler	NA	United Kingdom	2013	NA	Alba et al., 2020	1	0	1	1
KBAK-4500	ERR2218241	Food	Food	Meat from turkeys	NA	Romania	2016	NA	Alba et al., 2020	1	0	0	1
11-02782	ERR2200348	Animal	Animal	Turkey - fattening flocks	NA	Germany	2011	NA	Alba et al., 2020	1	0	0	1
13-SA00631	ERR2200351	Animal	Animal	Turkey - fattening flocks	NA	Germany	2013	NA	Alba et al., 2020	1	0	0	1
15-SA01362	ERR2200353	Food	Food	Meat from broilers	NA	Germany	2015	NA	Alba et al., 2020	1	0	0	1
16-SA00680	ERR2200362	Food	Food	Meat from broilers	NA	Germany	2016	NA	Alba et al., 2020	1	0	0	1
16-SA00911	ERR2200364	Food	Food	Meat from broilers	NA	Germany	2016	NA	Alba et al., 2020	1	0	0	1
17-SA00181	ERR2200381	Environment	Environment	Chicken	NA	Germany	2017	NA	Alba et al., 2020	1	0	0	1
14026835§	ERR1014119	Human	Human	Human	NA	NA	2014	America	Alba et al., 2020	1	0	1	1
2014AM-2863	SRR2353201	Human	Human	Human	NA	USA	2017	NA	Tate et al., 2017	1	0	1	1
N55391	SRR2407791	Food	Food	Meat from broilers	NA	USA	2017	NA	Tate et al., 2017	1	0	1	1
FSIS1504606	SRR2587646	Food	Food	Meat from broilers	NA	USA	2016	NA	Tate et al., 2017	1	0	1	1
FSIS1502973	SRR2939561	Animal	Animal	Cattle- dairy	NA	USA	2016	NA	Tate et al., 2017	1	0	1	1
FSIS1502169	SRR2939562	Animal	Animal	Cattle- dairy	NA	USA	2015	NA	Tate et al., 2017	1	0	1	1
FSIS1502967	SRR2939570	Food	Food	Meat from broilers	NA	USA	2014	NA	Tate et al., 2017	1	0	1	1
FSIS1502916 (reference)	SRR3129297	Food	Food	Meat from broilers	NA	USA	2015	NA	Tate et al., 2017	1	0	1	1
2013AM-0055	SRR4025935	Human	Human	Human	NA	USA	2013	NA	Tate et al., 2017	1	0	1	1
2013AM-1918	SRR4025936	Human	Human	Human	NA	USA	2013	NA	Tate et al., 2017	1	0	1	1

Table S4

Strain	Accession_number	Source	Sample_type	Host_species	Sampling_location	Country	Year	Foreign_travel	Source_article	IncFIB(pN55391)	IncI1-I(Alpha)	gyrA_D87Y	parC_T57S
2014AM-3028	SRR4025938	Human	Human	Human	NA	USA	2014	NA	Tate et al., 2017	1	0	1	1
N16S103	SRR6351071	Food	Food	Chicken breast	NA	USA	2016	NA	Tyson et al., 2021	1	0	1	1
N17S1509	SRR7653353	Food	Food	Chicken breast	NA	USA	2017	NA	Tyson et al., 2021	1	0	1	1
N16S097	SRR6350849	Food	Food	Chicken breast	NA	USA	2016	NA	Tyson et al., 2021	1	0	1	1
N16S024	SRR6350891	Food	Food	Chicken breast	NA	USA	2016	NA	Tyson et al., 2021	1	0	1	1
N19S0679	SRR10180524	Food	Food	Chicken wings	NA	USA	2019	NA	Tyson et al., 2021	1	0	1	1
N19S0388	SRR10180702	Food	Food	Chicken breast	NA	USA	2019	NA	Tyson et al., 2021	1	0	1	1
N19S0552	SRR10180585	Food	Food	Turkey	NA	USA	2019	NA	Tyson et al., 2021	1	0	1	1
N17S1040	SRR7653266	Food	Food	Chicken wings	NA	USA	2017	NA	Tyson et al., 2021	1	0	1	1
N17S1126	SRR8875071	Food	Food	ground turkey	NA	USA	2017	NA	Tyson et al., 2021	1	0	1	1
N18S2039	SRR9984225	Food	Food	ground turkey	NA	USA	2018	NA	Tyson et al., 2021	1	0	1	1
N17S1349	SRR7907764	Food	Food	Chicken wings	NA	USA	2017	NA	Tyson et al., 2021	1	0	1	1
N19S0611	SRR10180550	Food	Food	ground turkey	NA	USA	2019	NA	Tyson et al., 2021	1	0	1	1
N19S0641	SRR10180526	Food	Food	chicken legs	NA	USA	2019	NA	Tyson et al., 2021	1	0	1	1
N19S1233	SRR9917211	Food	Food	Turkey	NA	USA	2019	NA	Tyson et al., 2021	1	0	1	1
N17S1598	SRR7907778	Food	Food	Chicken breast	NA	USA	2017	NA	Tyson et al., 2021	1	0	1	1
SRR1965152	SRR1965152	Human	Faeces	Human	NA	UK	2014	Spain	Lee et al., 2021	1	0	0	1
SRR3049270	SRR3049270	Human	Faeces	Human	NA	UK	2014	Peru	Lee et al., 2021	1	0	1	1
SRR3049109	SRR3049109	Human	Faeces	Human	NA	UK	2014	Unknown	Lee et al., 2021	1	0	1	1
SRR1966556	SRR1966556	Human	Faeces	Human	NA	UK	2015	Peru	Lee et al., 2021	1	0	1	1
SRR6854755	SRR6854755	Human	Faeces	Human	NA	UK	2015	Unknown	Lee et al., 2021	1	0	1	1
SRR14780539	SRR14780539	Human	Faeces	Human	NA	UK	2015	Unknown	Lee et al., 2021	1	0	1	1
SRR6191309	SRR6191309	Human	Faeces	Human	NA	UK	2015	Unknown	Lee et al., 2021	1	0	1	1
SRR7298818	SRR7298818	Human	Faeces	Human	NA	UK	2015	Peru	Lee et al., 2021	1	0	1	1
SRR6193019	SRR6193019	Human	Faeces	Human	NA	UK	2015	Peru	Lee et al., 2021	1	0	1	1
SRR6191085	SRR6191085	Human	Faeces	Human	NA	UK	2015	Spain	Lee et al., 2021	1	0	0	1
SRR5193958	SRR5193958	Human	Faeces	Human	NA	UK	2016	Bolivia	Lee et al., 2021	1	0	1	1
SRR6237079	SRR6237079	Human	Faeces	Human	NA	UK	2017	Spain	Lee et al., 2021	1	0	0	1
SRR8258013	SRR8258013	Human	Blood	Human	NA	UK	2018	Unknown	Lee et al., 2021	1	0	1	1
SRR7997069	SRR7997069	Human	Faeces	Human	NA	UK	2018	Spain	Lee et al., 2021	1	0	1	1
SRR7873895	SRR7873895	Human	Faeces	Human	NA	UK	2018	Dominican republ	Lee et al., 2021	1	0	1	1
SRR8499050	SRR8499050	Human	Faeces	Human	NA	UK	2018	India	Lee et al., 2021	1	0	1	1
SRR8492342	SRR8492342	Food	Food	Chicken fillets	NA	UK	2018	Unknown	Lee et al., 2021	1	0	1	1
SRR8509076	SRR8509076	Food	Food	raw chicken breast	NA	UK	2018	Brazil	Lee et al., 2021	1	0	1	1
SRR7506922	SRR7506922	Food	Food	frozen chicken breast	NA	UK	2018	Chile	Lee et al., 2021	1	0	1	1
SRR7506433	SRR7506433	Food	Food	raw chicken breast	NA	UK	2018	Chile	Lee et al., 2021	1	0	1	1
SRR7507001	SRR7507001	Food	Food	raw chicken breast	NA	UK	2018	Chile	Lee et al., 2021	1	0	1	1
SRR7184567	SRR7184567	Human	Faeces	Human	NA	UK	2018	Unknown	Lee et al., 2021	1	0	1	1
SRR7501314	SRR7501314	Food	Food	Salted Chicken Breast	NA	UK	2018	Chile	Lee et al., 2021	1	0	1	1
SRR8508653	SRR8508653	Food	Food	Salted Chicken Breast	NA	UK	2018	Chile	Lee et al., 2021	1	0	1	1
SRR8523767	SRR8523767	Human	Faeces	Human	NA	UK	2018	Peru	Lee et al., 2021	1	0	1	1
G3A	SRR8266235	Animal	caeca content	Gallus gallus domesticus	NA	Ecuador (Galápagos	2016	NA	Burnett et al., 2021	1	0	1	1
G12A	SRR8266266	Animal	caeca content	Gallus gallus domesticus	NA	Ecuador (Galápagos	2016	NA	Burnett et al., 2021	1	0	1	1
G13A	SRR8266271	Animal	caeca content	Gallus gallus domesticus	NA	Ecuador (Galápagos	2016	NA	Burnett et al., 2021	1	0	1	1
G15A	SRR8266263	Animal	caeca content	Gallus gallus domesticus	NA	Ecuador (Galápagos	2016	NA	Burnett et al., 2021	1	0	1	1
LN649235.1	LN649235.1_Infantis	Animal	NA	Broiler chicken	NA	United Kingdom	1973	NA	Olasz et al., 2015				

Table S4

Strain	Accession_number	aac(6')-Iaa	aadA1	aph(4)-Ia	aac(3)-IV	aph(3')-Ia	blaCTX-M-65	floR	sul1	tet(A)	qacE	fosA3	dfrA14	blaTEM-1B	sul3	dfrA1	sul2	cmlA1	aadA13	aadA2b	qacG.x	aph(6)-Id	aph(3'')-Ib	blaCTX-M-1	mef(B)
ZTA22/01238	ERR12341388	1	0	0	0	0	0	0	0	0	0	0	0	0	0	0	0	0	0	0	0	0	0	0	0
06029746§	ERR1014111	1	1	0	0	0	0	0	0	0	0	0	0	0	1	0	0	1	1	1	0	0	0	0	0
SRR6190486	SRR6190486	1	0	0	0	0	0	0	0	0	0	0	0	0	0	0	0	0	0	0	0	0	0	0	0
SRR6191680	SRR6191680	1	0	0	0	0	0	0	0	0	0	0	0	0	0	0	0	0	0	0	0	0	0	0	0
SRR7298406	SRR7298406	1	0	0	0	0	0	0	0	0	0	0	0	0	0	0	0	0	0	0	0	0	0	0	0
SRR6233897	SRR6233897	1	0	0	0	0	0	0	0	0	0	0	0	0	0	0	0	0	0	0	0	0	0	0	0
SRR6237076	SRR6237076	1	0	0	0	0	0	0	0	0	0	0	0	0	0	0	0	0	0	0	0	0	0	0	0
SRR14774739	SRR14774739	1	0	0	0	0	0	0	0	0	0	0	0	0	0	0	0	0	0	0	0	0	0	0	0
ZTA22/01226	ERR12341384	1	1	1	1	1	1	1	1	1	1	0	0	0	0	0	0	0	0	0	0	0	0	0	0
ZTA22/01227	ERR12341385	1	1	1	1	0	0	0	1	1	1	1	1	0	0	0	0	0	0	0	0	0	0	0	0
ZTA22/01231	ERR12341386	1	1	0	0	0	0	0	1	1	1	0	1	0	0	0	0	0	0	0	0	0	0	0	0
ZTA22/01237	ERR12341387	1	1	1	1	1	1	1	1	1	1	1	1	0	0	0	0	0	0	0	0	0	0	0	0
ZTA22/01239	ERR12341389	1	1	1	1	1	1	1	1	1	1	1	1	0	0	0	0	0	0	0	0	0	0	0	0
ZTA22/01240	ERR12341390	1	1	1	1	1	1	1	1	1	1	1	1	0	0	0	0	0	0	0	0	0	0	0	0
ZTA22/01242	ERR12341391	1	1	1	1	1	1	1	1	1	1	1	1	0	0	0	0	0	0	0	0	0	0	0	0
1091603069	ERR2209370	1	1	1	1	1	1	1	1	1	1	1	1	0	0	0	0	0	0	0	0	0	0	0	0
1091600414	ERR2209376	1	1	1	1	1	1	0	1	1	1	1	1	0	0	0	0	0	0	0	0	0	0	0	0
1091300903	ERR2209379	1	1	1	1	0	1	1	1	1	1	1	1	0	0	0	0	0	0	0	1	0	0	0	0
1091400879	ERR2209381	1	1	1	1	0	1	1	1	1	1	1	1	0	0	0	0	0	0	0	0	0	0	0	0
1091401725	ERR2209388	1	1	1	1	0	1	1	1	1	1	1	1	0	0	0	0	0	0	0	0	0	0	0	0
17079324	ERR2209402	1	1	0	0	1	0	0	1	1	1	0	1	1	0	1	1	0	0	0	0	1	1	1	0
16066439	ERR2209412	1	1	0	0	0	0	0	1	1	1	0	0	1	1	1	0	1	0	1	0	0	0	1	0
16042038-1	ERR2209469	1	1	1	1	1	1	1	1	1	1	0	1	0	0	0	0	0	0	0	0	0	0	0	0
17022330-sal	ERR2209489	1	1	1	1	1	1	1	1	1	1	1	0	0	0	0	0	0	0	0	0	0	0	0	0
14040932-sal	ERR2209492	1	1	1	1	0	1	0	1	1	1	0	0	0	0	0	0	0	0	0	0	0	0	0	0
16082207	ERR2209609	1	1	0	0	1	0	0	1	1	1	0	1	0	0	0	0	0	0	0	0	0	0	0	0
13002124-23§	ERR2209663	1	0	0	0	1	0	0	1	1	1	0	1	0	1	1	0	0	0	0	0	0	0	1	1
36622§	ERR2209682	1	1	0	0	1	0	0	1	1	1	0	1	0	0	0	0	0	0	0	0	0	0	0	0
41175	ERR2209704	1	0	0	0	1	0	0	1	1	1	0	1	1	0	0	0	0	0	0	0	0	0	0	0
15078162-1	ERR3562263	1	1	0	0	0	0	0	1	1	1	0	0	0	0	0	0	0	0	0	0	0	0	0	0
L00458-17	ERR2231067	1	1	0	0	0	0	0	1	1	1	0	0	0	0	0	0	0	0	0	0	0	0	0	0
L01514-15	ERR2231037	1	1	0	0	0	0	0	1	1	1	0	0	0	0	0	0	0	0	0	0	0	0	0	0
S04446-15	ERR2231049	1	1	0	0	0	0	0	1	1	1	0	1	0	0	0	0	0	0	0	0	0	0	0	0
L02065-13	ERR2231046	1	1	0	0	0	0	0	1	1	1	0	1	0	0	0	0	0	0	0	0	0	0	0	0
KBAK-4500	ERR2218241	1	1	0	0	0	0	0	1	1	1	0	1	0	0	0	0	0	0	0	0	0	0	0	0
11-02782	ERR2200348	1	1	0	0	0	0	0	1	1	1	0	0	0	0	0	0	0	0	0	0	0	0	0	0
13-SA00631	ERR2200351	1	1	0	0	0	0	0	1	1	1	0	0	0	0	0	0	0	0	0	0	0	0	0	0
15-SA01362	ERR2200353	1	1	0	0	0	0	0	1	1	1	0	0	0	0	0	0	0	0	0	0	0	0	0	0
16-SA00680	ERR2200362	1	1	0	0	1	0	0	1	1	1	0	1	1	0	0	0	0	0	0	0	0	0	0	0
16-SA00911	ERR2200364	1	1	0	0	0	0	0	1	1	1	0	1	0	0	0	0	0	0	0	0	0	0	0	0
17-SA00181	ERR2200381	1	1	0	0	0	0	0	1	1	1	0	0	0	0	0	0	0	0	0	0	0	0	0	0
14026835§	ERR1014119	1	1	1	1	1	1	1	1	1	1	1	1	0	0	0	0	0	0	0	0	0	0	0	0
2014AM-2863	SRR2353201	1	1	1	1	1	1	1	1	1	1	1	1	0	0	0	0	0	0	0	0	0	0	0	0
N55391	SRR2407791	1	0	1	1	1	1	1	1	1	0	0	1	0	0	0	0	0	0	0	0	0	0	0	0
FSIS1504606	SRR2587646	1	1	1	1	1	1	0	1	1	1	1	1	0	0	0	0	0	0	0	0	0	0	0	0
FSIS1502973	SRR2939561	1	1	1	1	0	0	1	1	1	1	0	0	0	0	0	0	0	0	0	0	0	0	0	0
FSIS1502169	SRR2939562	1	1	1	1	1	1	1	1	1	1	1	1	0	0	0	0	0	0	0	0	0	0	0	0
FSIS1502967	SRR2939570	1	1	1	1	1	1	1	1	1	1	1	1	0	0	0	0	0	0	0	0	0	0	0	0
FSIS1502916 (reference)	SRR3129297	1	1	1	1	1	1	1	1	1	1	1	1	0	0	0	0	0	0	0	0	0	0	0	0
2013AM-0055	SRR4025935	1	1	1	1	0	1	1	1	1	1	1	1	0	0	0	0	0	0	0	0	0	0	0	0
2013AM-1918	SRR4025936	1	1	1	1	1	1	1	1	1	1	0	1	0	0	0	0	0	0	0	0	0	0	0	0

Table S4

Strain	Accession_number	aac(6')-Iaa	aadA1	aph(4)-Ia	aac(3)-IV	aph(3')-Ia	blaCTX-M-65	floR	sul1	tet(A)	qacE	fosA3	dfrA14	blaTEM-1B	sul3	dfrA1	sul2	cmlA1	aadA13	aadA2b	qacG.x	aph(6)-Id	aph(3'')-Ib	blaCTX-M-1	mef(B)
2014AM-3028	SRR4025938	1	1	1	1	0	1	1	1	1	1	0	1	0	0	0	0	0	0	0	0	0	0	0	0
N16S103	SRR6351071	1	1	1	1	1	1	1	1	0	1	1	1	0	0	0	0	0	0	0	0	0	0	0	0
N17S1509	SRR7653353	1	1	1	1	1	0	1	1	1	1	1	1	0	0	0	0	0	0	0	0	0	0	0	0
N16S097	SRR6350849	1	1	1	1	1	1	1	1	1	1	0	1	0	0	0	0	0	0	0	0	0	0	0	0
N16S024	SRR6350891	1	1	1	1	1	1	1	1	1	1	1	1	0	0	0	0	0	0	0	0	0	0	0	0
N19S0679	SRR10180524	1	1	1	1	1	1	1	1	1	1	1	0	0	0	0	0	0	0	0	0	0	0	0	0
N19S0388	SRR10180702	1	1	0	0	0	1	0	1	1	1	0	0	0	0	0	0	0	0	0	0	0	0	0	0
N19S0552	SRR10180585	1	1	0	0	0	1	0	1	1	1	0	0	1	0	0	0	0	0	0	0	0	0	0	0
N17S1040	SRR7653266	1	1	1	1	0	0	1	1	1	1	0	1	0	0	0	0	0	0	0	0	0	0	0	0
N17S1126	SRR8875071	1	1	1	1	1	1	1	1	1	1	1	1	0	0	0	0	0	0	0	0	0	0	0	0
N18S2039	SRR9984225	1	0	0	0	0	1	0	0	1	0	0	0	0	0	0	0	0	0	0	0	0	0	0	0
N17S1349	SRR7907764	1	1	1	1	1	0	0	1	1	1	0	0	0	0	0	0	0	0	0	0	0	0	0	0
N19S0611	SRR10180550	1	1	1	1	1	1	1	1	1	1	1	1	0	0	0	0	0	0	0	0	0	0	0	0
N19S0641	SRR10180526	1	1	1	1	0	1	1	1	1	1	1	1	0	0	0	0	0	0	0	0	0	0	0	0
N19S1233	SRR9917211	1	1	1	1	1	1	0	1	1	1	1	1	0	0	0	0	0	0	0	0	0	0	0	0
N17S1598	SRR7907778	1	1	1	1	0	1	1	1	1	1	0	0	0	0	0	0	0	0	0	0	0	0	0	0
SRR1965152	SRR1965152	1	1	0	0	0	0	0	1	1	1	0	0	0	0	0	0	0	0	0	0	0	0	0	0
SRR3049270	SRR3049270	1	1	1	1	1	1	1	1	1	1	0	1	0	0	0	0	0	0	0	0	0	0	0	0
SRR3049109	SRR3049109	1	1	1	1	0	1	1	1	1	1	0	1	0	0	0	0	0	0	0	0	0	0	0	0
SRR1966556	SRR1966556	1	1	1	1	1	1	0	1	1	1	1	1	0	0	0	0	0	0	0	0	0	0	0	0
SRR6854755	SRR6854755	1	1	1	1	1	1	1	1	1	1	1	1	0	0	0	0	0	0	0	0	0	0	0	0
SRR14780539	SRR14780539	1	1	1	1	1	1	1	1	1	1	1	1	0	0	0	0	0	0	0	0	0	0	0	0
SRR6191309	SRR6191309	1	0	1	1	1	1	1	0	0	0	1	1	0	0	0	0	0	0	0	0	0	0	0	0
SRR7298818	SRR7298818	1	1	1	1	1	1	1	1	1	1	0	1	0	0	0	0	0	0	0	0	0	0	0	0
SRR6193019	SRR6193019	1	1	1	1	1	1	1	1	1	1	1	1	0	0	0	0	0	0	0	0	0	0	0	0
SRR6191085	SRR6191085	1	1	0	0	0	0	0	1	1	1	0	0	0	0	0	0	0	0	0	0	0	0	0	0
SRR5193958	SRR5193958	1	1	1	1	1	1	1	1	1	1	1	1	0	0	0	0	0	0	0	0	0	0	0	0
SRR6237079	SRR6237079	1	1	0	0	0	0	0	1	1	1	0	0	0	0	0	0	0	0	0	0	0	0	0	0
SRR8258013	SRR8258013	1	1	1	1	1	1	1	1	1	1	1	1	0	0	0	0	0	0	0	0	0	0	0	0
SRR7997069	SRR7997069	1	1	1	1	1	1	1	1	1	1	1	1	0	0	0	0	0	0	0	0	0	0	0	0
SRR7873895	SRR7873895	1	1	1	1	1	1	1	1	1	1	1	1	0	0	0	0	0	0	0	0	0	0	0	0
SRR8499050	SRR8499050	1	1	1	1	1	1	1	1	1	1	0	1	0	0	0	0	0	0	0	0	0	0	0	0
SRR8492342	SRR8492342	1	1	1	1	1	1	1	1	1	1	1	1	0	0	0	0	0	0	0	0	0	0	0	0
SRR8509076	SRR8509076	1	1	1	1	1	1	1	1	1	1	0	1	0	0	0	0	0	0	0	0	0	0	0	0
SRR7506922	SRR7506922	1	1	1	1	1	1	1	1	1	1	1	1	0	0	0	0	0	0	0	0	0	0	0	0
SRR7506433	SRR7506433	1	1	1	1	1	1	1	1	1	1	1	1	0	0	0	0	0	0	0	0	0	0	0	0
SRR7507001	SRR7507001	1	1	1	1	1	1	1	1	1	1	0	1	0	0	0	0	0	0	0	0	0	0	0	0
SRR7184567	SRR7184567	1	1	1	1	1	1	1	1	1	1	0	1	0	0	0	0	0	0	0	0	0	0	0	0
SRR7501314	SRR7501314	1	1	1	1	0	1	1	1	1	1	0	0	0	0	0	0	0	0	0	0	0	0	0	0
SRR8508653	SRR8508653	1	1	1	1	0	1	1	1	1	1	0	0	0	0	0	0	0	0	0	0	0	0	0	0
SRR8523767	SRR8523767	1	1	1	1	1	1	1	1	1	1	1	1	0	0	0	0	0	0	0	0	0	0	0	0
G3A	SRR8266235	1	1	1	1	1	0	1	1	1	1	0	1	0	0	0	0	0	0	0	0	0	0	0	0
G12A	SRR8266266	1	1	1	1	0	1	1	1	1	1	0	1	0	0	0	0	0	0	0	0	0	0	0	0
G13A	SRR8266271	1	1	1	1	1	1	1	1	1	1	1	1	0	0	0	0	0	0	0	0	0	0	0	0
G15A	SRR8266263	1	1	1	1	0	1	1	1	1	1	0	1	0	0	0	0	0	0	0	0	0	0	0	0
LN649235.1	LN649235.1_Infantis																								

Table S4

Strain	Accession_number	lnu(G)	golT	golS	arsR	iroB	iroC	sinH	ybtP	ybtQ	ybtE	ybtT	ybtU	ybtA	ybtX	ybtS	irp1	irp2	fyuA/psn	merR	merT	merP	merC	qacEdelta1
ZTA22/01238	ERR12341388	0	1	1	1	1	1	1	0	0	0	0	0	0	0	0	0	0	0	0	0	0	0	0
06029746§	ERR1014111	0	1	1	1	1	1	1	0	0	0	0	0	0	0	0	0	0	0	0	0	0	0	0
SRR6190486	SRR6190486	0	1	1	1	1	1	1	0	0	0	0	0	0	0	0	0	0	0	0	0	0	0	0
SRR6191680	SRR6191680	0	1	1	1	1	1	1	0	0	0	0	0	0	0	0	0	0	0	0	0	0	0	0
SRR7298406	SRR7298406	0	1	1	1	1	1	1	0	0	0	0	0	0	0	0	0	0	0	0	0	0	0	0
SRR6233897	SRR6233897	0	1	1	1	1	1	1	0	0	0	0	0	0	0	0	0	0	0	0	0	0	0	0
SRR6237076	SRR6237076	0	1	1	1	1	1	1	0	0	0	0	0	0	0	0	0	0	0	0	0	0	0	0
SRR14774739	SRR14774739	0	1	1	1	1	1	1	0	0	0	0	0	0	0	0	0	0	0	0	0	0	0	0
ZTA22/01226	ERR12341384	0	1	1	1	1	1	1	1	1	1	1	1	1	1	1	1	1	1	1	1	1	1	1
ZTA22/01227	ERR12341385	0	1	1	1	1	1	1	1	1	1	1	1	1	1	1	1	1	1	1	1	1	1	1
ZTA22/01231	ERR12341386	0	1	1	1	1	1	1	1	1	1	1	1	1	1	1	1	1	1	1	1	1	1	1
ZTA22/01237	ERR12341387	0	1	1	1	1	1	1	1	1	1	1	1	1	1	1	1	1	1	1	1	1	1	1
ZTA22/01239	ERR12341389	0	1	1	1	1	1	1	1	1	1	1	1	1	1	1	1	1	1	1	1	1	1	1
ZTA22/01240	ERR12341390	0	1	1	1	1	1	1	1	1	1	1	1	1	1	1	1	1	1	1	1	1	1	1
ZTA22/01242	ERR12341391	0	1	1	1	1	1	1	1	1	1	1	1	1	1	1	1	1	1	1	1	1	1	1
1091603069	ERR2209370	0	1	1	1	1	1	1	1	1	1	1	1	1	1	1	1	1	1	1	1	1	1	1
1091600414	ERR2209376	0	1	1	1	1	1	1	1	1	1	1	1	1	1	1	1	1	1	1	1	1	1	1
1091300903	ERR2209379	0	1	1	1	1	1	1	1	1	1	1	1	1	1	1	1	1	1	1	1	1	1	1
1091400879	ERR2209381	0	1	1	1	1	1	1	1	1	1	1	1	1	1	1	1	1	1	1	1	1	1	1
1091401725	ERR2209388	0	1	1	1	1	1	1	1	1	1	1	1	1	1	1	1	1	1	1	1	1	1	1
17079324	ERR2209402	0	1	1	1	1	1	1	1	1	1	1	1	1	1	1	1	1	1	1	1	1	1	1
16066439	ERR2209412	0	1	1	1	1	1	1	1	1	1	1	1	1	1	1	1	1	1	1	1	1	1	1
16042038-1	ERR2209469	0	1	1	1	1	1	1	1	1	1	1	1	1	1	1	1	1	1	1	1	1	1	1
17022330-sal	ERR2209489	0	1	1	1	1	1	1	1	1	1	1	1	1	1	1	1	1	1	1	1	1	1	1
14040932-sal	ERR2209492	0	1	1	1	1	1	1	1	1	1	1	1	1	1	1	1	1	1	1	1	1	1	1
16082207	ERR2209609	0	1	1	1	1	1	1	1	1	1	1	1	1	1	1	1	1	1	1	1	1	1	1
13002124-23§	ERR2209663	0	1	1	1	1	1	1	1	1	1	1	1	1	1	1	1	1	1	1	1	1	1	1
36622§	ERR2209682	0	1	1	1	1	1	1	1	1	1	1	1	1	1	1	1	1	1	1	1	1	1	1
41175	ERR2209704	1	1	1	1	1	1	1	1	1	1	1	1	1	1	1	1	1	1	1	1	1	1	1
15078162-1	ERR3562263	0	1	1	1	1	1	1	1	1	1	1	1	1	1	1	1	1	1	1	1	1	1	1
L00458-17	ERR2231067	0	1	1	1	1	1	1	1	1	1	1	1	1	1	1	1	1	1	1	1	1	1	1
L01514-15	ERR2231037	0	1	1	1	1	1	1	1	1	1	1	1	1	1	1	1	1	1	1	1	1	1	1
S04446-15	ERR2231049	0	1	1	1	1	1	1	1	1	1	1	1	1	1	1	1	1	1	1	1	1	1	1
L02065-13	ERR2231046	0	1	1	1	1	1	1	1	1	1	1	1	1	1	1	1	1	1	1	1	1	1	1
KBAK-4500	ERR2218241	0	1	1	1	1	1	1	1	1	1	1	1	1	1	1	1	1	1	1	1	1	1	1
11-02782	ERR2200348	0	1	1	1	1	1	1	1	1	1	1	1	1	1	1	1	1	1	1	1	1	1	1
13-SA00631	ERR2200351	0	1	1	1	1	1	1	1	1	1	1	1	1	1	1	1	1	1	1	1	1	1	1
15-SA01362	ERR2200353	0	1	1	1	1	1	1	1	1	1	1	1	1	1	1	1	1	1	1	1	1	1	1
16-SA00680	ERR2200362	0	1	1	1	1	1	1	1	1	1	1	1	1	1	1	1	1	1	1	1	1	1	1
16-SA00911	ERR2200364	0	1	1	1	1	1	1	1	1	1	1	1	1	1	1	1	1	1	1	1	1	1	1
17-SA00181	ERR2200381	0	1	1	1	1	1	1	1	1	1	1	1	1	1	1	1	1	1	1	1	1	1	1
14026835§	ERR1014119	0	1	1	1	1	1	1	1	1	1	1	1	1	1	1	1	1	1	1	1	1	1	1
2014AM-2863	SRR2353201	0	1	1	1	1	1	1	1	1	1	1	1	1	1	1	1	1	1	1	1	1	1	1
N55391	SRR2407791	0	1	1	1	1	1	1	1	1	1	1	1	1	1	1	1	1	1	1	1	1	1	0
FSIS1504606	SRR2587646	0	1	1	1	1	1	1	1	1	1	1	1	1	1	1	1	1	1	1	1	1	1	1
FSIS1502973	SRR2939561	0	1	1	1	1	1	1	1	1	1	1	1	1	1	1	1	1	1	1	1	1	1	1
FSIS1502169	SRR2939562	0	1	1	1	1	1	1	1	1	1	1	1	1	1	1	1	1	1	1	1	1	1	1
FSIS1502967	SRR2939570	0	1	1	1	1	1	1	1	1	1	1	1	1	1	1	1	1	1	1	1	1	1	1
FSIS1502916 (reference)	SRR3129297	0	1	1	1	1	1	1	1	1	1	1	1	1	1	1	1	1	1	1	1	1	1	1
2013AM-0055	SRR4025935	0	1	1	1	1	1	1	1	1	1	1	1	1	1	1	1	1	1	1	1	1	1	1
2013AM-1918	SRR4025936	0	1	1	1	1	1	1	1	1	1	1	1	1	1	1	1	1	1	1	1	1	1	1

Table S4

Strain	Accession_number	lnu(G)	golT	golS	arsR	iroB	iroC	sinH	ybtP	ybtQ	ybtE	ybtT	ybtU	ybtA	ybtX	ybtS	irp1	irp2	fyuA/psn	merR	merT	merP	merC	qacEdelta1
2014AM-3028	SRR4025938	0	1	1	1	1	1	1	1	1	1	1	1	1	1	1	1	1	1	1	1	1	1	1
N16S103	SRR6351071	0	1	1	1	1	1	1	1	1	1	1	1	1	1	1	1	1	1	1	1	1	1	1
N17S1509	SRR7653353	0	1	1	1	1	1	1	1	1	1	1	1	1	1	1	1	1	1	1	1	1	1	1
N16S097	SRR6350849	0	1	1	1	1	1	1	1	1	1	1	1	1	1	1	1	1	1	1	1	1	1	1
N16S024	SRR6350891	0	1	1	1	1	1	1	1	1	1	1	1	1	1	1	1	1	1	1	1	1	1	1
N19S0679	SRR10180524	0	1	1	1	1	1	1	1	1	1	1	1	1	1	1	1	1	1	1	1	1	1	1
N19S0388	SRR10180702	0	1	1	1	1	1	1	1	1	1	1	1	1	1	1	1	1	1	1	1	1	1	1
N19S0552	SRR10180585	0	1	1	1	1	1	1	1	1	1	1	1	1	1	1	1	1	1	1	1	1	1	1
N17S1040	SRR7653266	0	1	1	1	1	1	1	1	1	1	1	1	1	1	1	1	1	1	1	1	1	1	1
N17S1126	SRR8875071	0	1	1	1	1	1	1	1	1	1	1	1	1	1	1	1	1	1	1	1	1	1	1
N18S2039	SRR9984225	0	1	1	1	1	1	1	1	1	1	1	1	1	1	1	1	1	1	1	1	1	1	0
N17S1349	SRR7907764	0	1	1	1	1	1	1	1	1	1	1	1	1	1	1	1	1	1	1	1	1	1	1
N19S0611	SRR10180550	0	1	1	1	1	1	1	1	1	1	1	1	1	1	1	1	1	1	1	1	1	1	1
N19S0641	SRR10180526	0	1	1	1	1	1	1	1	1	1	1	1	1	1	1	1	1	1	1	1	1	1	1
N19S1233	SRR9917211	0	1	1	1	1	1	1	1	1	1	1	1	1	1	1	1	1	1	1	1	1	1	1
N17S1598	SRR7907778	0	1	1	1	1	1	1	1	1	1	1	1	1	1	1	1	1	1	1	1	1	1	1
SRR1965152	SRR1965152	0	1	1	1	1	1	1	1	1	1	1	1	1	1	1	1	1	1	1	1	1	1	1
SRR3049270	SRR3049270	0	1	1	1	1	1	1	1	1	1	1	1	1	1	1	1	1	1	1	1	1	1	1
SRR3049109	SRR3049109	0	1	1	1	1	1	1	1	1	1	1	1	1	1	1	1	1	1	1	1	1	1	1
SRR1966556	SRR1966556	0	1	1	1	1	1	1	1	1	1	1	1	1	1	1	1	1	1	1	1	1	1	1
SRR6854755	SRR6854755	0	1	1	1	1	1	1	1	1	1	1	1	1	1	1	1	1	1	1	1	1	1	1
SRR14780539	SRR14780539	0	1	1	1	1	1	1	1	1	1	1	1	1	1	1	1	1	1	1	1	1	1	1
SRR6191309	SRR6191309	0	1	1	1	1	1	1	1	1	1	1	1	1	1	1	1	1	1	0	0	0	0	0
SRR7298818	SRR7298818	0	1	1	1	1	1	1	1	1	1	1	1	1	1	1	1	1	1	1	1	1	1	1
SRR6193019	SRR6193019	0	1	1	1	1	1	1	1	1	1	1	1	1	1	1	1	1	1	1	1	1	1	1
SRR6191085	SRR6191085	0	1	1	1	1	1	1	1	1	1	1	1	1	1	1	1	1	1	1	1	1	1	1
SRR5193958	SRR5193958	0	1	1	1	1	1	1	1	1	1	1	1	1	1	1	1	1	1	1	1	1	1	1
SRR6237079	SRR6237079	0	1	1	1	1	1	1	1	1	1	1	1	1	1	1	1	1	1	1	1	1	1	1
SRR8258013	SRR8258013	0	1	1	1	1	1	1	1	1	1	1	1	1	1	1	1	1	1	1	1	1	1	1
SRR7997069	SRR7997069	0	1	1	1	1	1	1	1	1	1	1	1	1	1	1	1	1	1	1	1	1	1	1
SRR7873895	SRR7873895	0	1	1	1	1	1	1	1	1	1	1	1	1	1	1	1	1	1	1	1	1	1	1
SRR8499050	SRR8499050	0	1	1	1	1	1	1	1	1	1	1	1	1	1	1	1	1	1	1	1	1	1	1
SRR8492342	SRR8492342	0	1	1	1	1	1	1	1	1	1	1	1	1	1	1	1	1	1	1	1	1	1	1
SRR8509076	SRR8509076	0	1	1	1	1	1	1	1	1	1	1	1	1	1	1	1	1	1	1	1	1	1	1
SRR7506922	SRR7506922	0	1	1	1	1	1	1	1	1	1	1	1	1	1	1	1	1	1	1	1	1	1	1
SRR7506433	SRR7506433	0	1	1	1	1	1	1	1	1	1	1	1	1	1	1	1	1	1	1	1	1	1	1
SRR7507001	SRR7507001	0	1	1	1	1	1	1	1	1	1	1	1	1	1	1	1	1	1	1	1	1	1	1
SRR7184567	SRR7184567	0	1	1	1	1	1	1	1	1	1	1	1	1	1	1	1	1	1	1	1	1	1	1
SRR7501314	SRR7501314	0	1	1	1	1	1	1	1	1	1	1	1	1	1	1	1	1	1	1	1	1	1	1
SRR8508653	SRR8508653	0	1	1	1	1	1	1	1	1	1	1	1	1	1	1	1	1	1	1	1	1	1	1
SRR8523767	SRR8523767	0	1	1	1	1	1	1	1	1	1	1	1	1	1	1	1	1	1	1	1	1	1	1
G3A	SRR8266235	0	1	1	1	1	1	1	1	1	1	1	1	1	1	1	1	1	1	1	1	1	1	1
G12A	SRR8266266	0	1	1	1	1	1	1	1	1	1	1	1	1	1	1	1	1	1	1	1	1	1	1
G13A	SRR8266271	0	1	1	1	1	1	1	1	1	1	1	1	1	1	1	1	1	1	1	1	1	1	1
G15A	SRR8266263	0	1	1	1	1	1	1	1	1	1	1	1	1	1	1	1	1	1	1	1	1	1	1
LN649235.1	LN649235.1_Infantis																							
